# An Energy-Efficient Unselfish Spectrum Leasing Scheme for Cognitive Radio Networks

**DOI:** 10.3390/s20216161

**Published:** 2020-10-29

**Authors:** Denis Bilibashi, Enrico M. Vitucci, Vittorio Degli-Esposti, Andrea Giorgetti

**Affiliations:** Department of Electrical, Electronic, and Information Engineering “Guglielmo Marconi” (DEI), CNIT, University of Bologna, 40126 Bologna, Italy; denis.bilibashi2@unibo.it (D.B.); enricomaria.vitucci@unibo.it (E.M.V.); v.degliesposti@unibo.it (V.D.-E.)

**Keywords:** cognitive radio, convex optimization, energy efficiency, power allocation, relay selection, spectrum leasing

## Abstract

Cooperative Communications in Cognitive Radio (CR) have been introduced as an essential and efficient technique to improve the transmission performance of primary users and offer transmission opportunities for secondary users. In a typical multiuser Cooperative Communication in CR, each primary user can choose one secondary user as a relay node. To encourage the cooperative behavior of the secondary users, primary users lease a fraction of their allocated spectrum to the relay secondary users to transmit their data packets. In this work, a novel unselfish spectrum leasing scheme in CR networks is proposed that offers an energy-efficient solution minimizing the environmental impact of our network. A network management architecture is introduced, and resource allocation is proposed as a constrained sum energy efficiency maximization problem. The optimization problem is formulated and solved using non-linear programming methods and based on a modified Kuhn-Munkres bipartite matching algorithm. System simulations demonstrate an increment in the energy efficiency of the primary users’ network compared with previously proposed algorithms.

## 1. Introduction

The exponential growth of wireless services in the last decades has called for additional spectrum to accommodate the ever-increasing traffic flow [1]. Besides, the current static spectrum policy, used by most countries in the world, has almost resulted in licensed spectrum exhaustion of some frequency bands [2]. On the contrary, recent spectrum utilization measurements have shown that the available spectrum opportunities are severely underutilized [3]. Cognitive radio (CR), with its ability to sense available frequency bands and adaptively adjust transmission frequency, has attracted considerable attention as one of the most promising solutions to spectrum underutilization [4,5,6,7]. Traditionally in CR networks (CRNs), there two categories of users, namely, primary users (PUs) and secondary users (SUs). PUs are licensed users, and they have the exclusive right to use their respective channels, while SUs are unlicensed, and they wish to use the underutilized channels [8]. To pay off the use of a licensed band, cooperative CRNs (CCRNs) have been proposed in which SUs act as relays to provide a better transmission performance to the PUs [9,10,11].

Extensive research has been conducted regarding spectrum leasing in CCRN. To address the challenges several approaches have been adopted. The game-theoretical approaches, such as Stackelberg game [12], are used to achieve the equilibrium state (e.g., Nash Equilibrium [13,14]) and involves PUs and SUs as players of the game. In Reference [15], a scenario, in which PUs compete for cooperation with available SUs under shared constraint set by SU’s requirements, is modeled as a generalized Nash Equilibrium. The authors of Reference [16], propose a noncooperative game to find the optimal power of SUs consumed in a scenario where SUs are used as relays in cooperation to aid PUs’ transmission. In Reference [17], a spectrum leasing technique to improve the spectral utility of the PUs has been proposed. Nash bargaining is used to determine the optimal division of temporal resources between relaying and reimbursement. In Reference [18], a novel approach is presented in which the spectrum leasing problem is formulated as a Stackelberg game, while the relay selection is solved by the Hungarian method. The authors of Reference [19], derive a cooperative game to find the optimal spectrum leasing time and allocated power of SUs.

Another popular approach is convex optimization. An adaptive spectrum leasing scheme is proposed in Reference [20] to improve the overall network throughput. The authors of Reference [21], present a spectrum leasing scheme for an overlay CRN in which PUs’ transmission is assisted by a cognitive multi-hop multi-branch network. The branch selection and spectrum allocation are jointly optimized such that the sum-rate of the SUs is maximized. A multi-objective optimization framework to incorporate average sum power minimization and the leased time minimization is proposed in Reference [22]. In Reference [23], a cooperative sharing model for cognitive wireless powered networks is proposed, to accomplish PU’s transmission early and vacate opportunity for SU’s transmission. An energy efficiency and spectral efficiency trade-off in an energy harvesting cooperative CRN is addressed in Reference [24]. The objective is to maximize energy and spectral efficiency via joint optimization of spectrum sensing time and self as well as cooperative transmission gains.

Different other approaches have been chosen to address the challenges of spectrum leasing in CCRN. In Reference [25], an opportunistic relaying based spectrum leasing is presented in which PUs lease their frequency band to the SUs and, at the same time, the primary network benefits in terms of outage probability. A cooperative relaying scheme is investigated in Reference [26], where one of the inactive SUs is used as a relay for the PUs. By adopting an aspiration level to model SUs behavior, the SUs’ strategy dynamics is formulated as a discrete-time Markov chain [27].

However, the majority of the research works regarding CRNs, are considered mostly PU-centered, with PUs that decide if and when to exploit SUs as relays based on certain conditions that can improve PUs’ transmission. This approach cannot guarantee full cooperation between PUs and SUs because will be dominated by the selfish behavior of PUs. In addition, networks are required nowadays to be more efficient not only in terms of spectrum use but also in terms of optimal power while guaranteeing a satisfying level of quality of service (QoS). In References [15,25,26], a scenario where a single pair of PUs or SUs is considered. Single user transmissions in CCRN compared to multiuser ones, can result incomplete because a full description of the efficiency of a cell cannot be given. It is proven that the rising number of users in a CCRN can significantly affect the overall performance of the network. Furthermore, spectrum leasing schemes in which PUs or SUs are assumed to have fixed transmission power like in Reference [18], cannot fully exploit the possibility of a more energy efficient network. Besides, energy-efficient systems are required not only from an engineering point of view but also to decrease the environmental impact through networks with a low carbon footprint [28].

Motivated by the aforementioned observations, we aim at jointly minimize the overall power consumption for energy-efficient communication in multiuser CRN. In addition, to provide improved cooperation between PUs and SUs and to secure an unselfish behavior of each user, we propose a resource management network architecture for a considered cell. This architecture, composed of various entities, will be responsible for making decisions regarding the usage of relay and for applying the optimal strategy to achieve the maximum energy efficiency of the cell. Our proposed CRN consists of multiple PUs and multiple SUs in which SUs are used as relays to enhance the PUs’ transmission performance and, in return, PUs lease a fraction of their bandwidth to the corresponding SUs. We formulate an energy efficiency based resource allocation, power allocation, and relay selection as an optimization problem. Our analysis shows that this problem can be divided into two subproblems: (1) power allocation, (2) transmission mode and relay selection. To solve the power allocation subproblem, we choose the maximization of the sum of energy efficiencies as the optimization target. This method was initially introduced in Reference [29] but due to its sum-of-ratio form can become difficult to solve. The method is further developed in our work by transforming power allocation into a subtractive form optimization problem with methods similar to those used to solve classical fractional programming problems [30,31,32]. Therefore, the optimal powers closed-form expressions can be derived through a two-layer optimization. Furthermore, the transmission mode and relay selection subproblem is solved based on a modified Kuhn-Munkres bipartite matching algorithm. System simulations performed in a referenced multiuser case validate the effectiveness of our approach proven by the proposed algorithm’s convergence and the increment in the efficiency of the PUs’ network performance when channel conditions get worse.

To summarize, the main contributions of the paper are:

- A novel unselfish resource allocation scheme for CRNs. Unlike most of works where PUs decide whether or not to cooperate with SUs, the decisions are made by a centralized approach with the goal of improving the overall energy efficiency of the cell.
- A resource management network architecture responsible for maximizing the energy efficiency of the cell.
- We propose a two-stage three-dimensional matching algorithm to maximize the energy efficiency of the cell, in which the joint optimization problem is decoupled into two subproblems and solved separately in two stages.

The rest of this paper is organized as follows. In Section 2, the architecture and the system model for CCRN are introduced. In Section 3, we formulate the energy efficiency based resource allocation, power allocation, and relay selection as an optimization problem. The non-convex energy efficiency for CCRN optimization problem is formulated and solved via an iterative algorithm in Section 4. Section 5 presents numerical performance results, and finally, conclusions are provided in Section 5.

## 2. Proposed Architecture and System Model for CCRN

**Figure 1:**
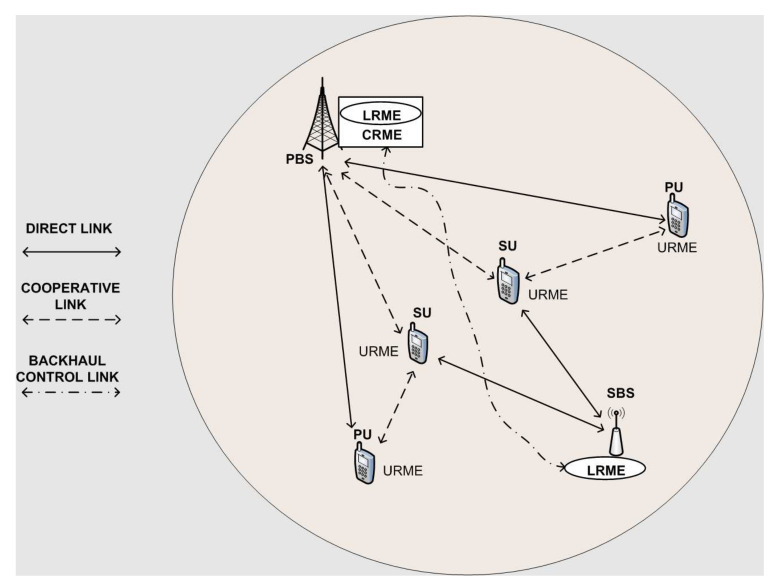
Proposed architecture and scenario for the cell.

In CCRN, one of the goals is to use the available spectrum resources in an efficient and coordinated way to guarantee a satisfying level of QoS for all users and achieve performance enhancement of the whole network. To this end, in this section, we propose a resource management network architecture consisting of a number of user resource management entities (URMEs), local resource management entities (LRMEs), and one cell resource management entity (CRME), as shown in Figure 1. The main functions of UMREs, LRMEs, and CRME are described as follows.

URME: It is a functional module embedded in each PU and SU, used to store channel state information, device characteristics and service requirements, and so forth. Through contacting associated LRMEs, URMEs send the collected information to the LRMEs and receive the resource allocation strategy accordingly also from CRMEs through LRMEs.

LRME: It is deployed in each primary base station (PBS) or secondary base station (SBS), being responsible for managing local resource status through interacting with the associated URMEs and the CRME. More specifically, receiving the network and service information from URMEs and then forwarding to the CRME, and receiving the resource management strategy from the CRME and forwarding to the URMEs.

CRME: By interacting with the LRME, the CMRE receives the network status, channel state information, and user service requirement information of all the users within the cell; then performs the proposed resource allocation and relay selection algorithm to obtain the optimal strategy for the cell and communicated to the associated LRMEs.

### System Model

In this paper, we consider a CCRN consisting of multiple PUs, multiple SUs, one PBS, and one SBS. Assume PUs are allowed to transmit to the PBS simultaneously, by orthogonal frequency division multiple access (OFDMA). Further, assume that PUs may transmit to the PBS in direct transmission mode or one-hop relay transmission mode, while the relay SUs may also transmit their own data packets to the SBS. Figure 1 illustrates the scenario considered in this paper.

To encourage SUs to relay data packets for the PUs, we propose a spectrum leasing scheme where the PUs lease part of their allocated spectrum to relay SUs so that they can transmit their own data to the SBS exploiting the licensed spectrum [33,34,35]. In this paper, we assume that the decode-and-forward (DF) scheme is applied at each relay node [36].

**Figure 2:**
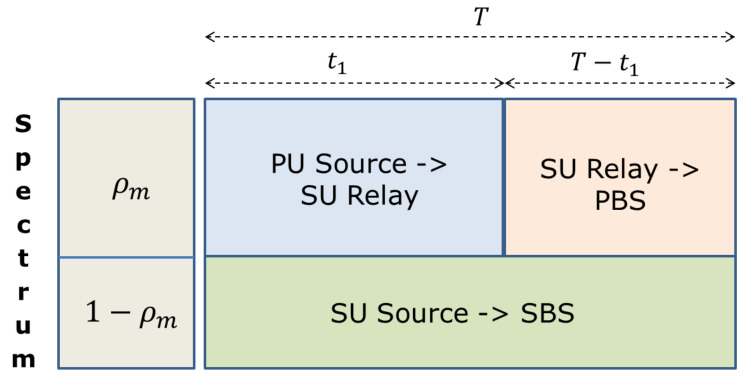
Bandwidth allocation and time slot division for primary user (PU) and secondary user (SU).

Let *M* and *K* denote the number of PUs and the number of SUs, respectively, $B_{m}=\rho_{m}B$ denote the allocated bandwidth of the *m*th PU, m=1,…,M, where $\rho_{m}\in \left[0,1\right]$ denotes the spectrum fraction of the *m*th PU for transmitting the data packets of the PU in the relay transmission mode, and as a consequence $B_{k}=\left(1-\rho_{m}\right)B$, ∀k, is the remaining spectrum available for the relay SU to transmit its own data to the SBS. To support relay communication, the transmission time slot *T* is divided into two periods. For the first part, the PUs transmit their data packets to the corresponding relay SUs, then the SUs forward the received data packets to the PBS during the remaining time. Meanwhile, the SUs transmit their own data packets to the SBS for the whole time period. Figure 2 shows the time and spectrum division mode for relay transmission of the *m*th PU.

We assume, all channels undergo flat Rayleigh fading and log-normal shadowing and we consider that LRMEs know all channel state information. The distance between *m*th PU and *k*th SU relay is denoted as $d_{m,k}^{\left(p,s\right)}$. Then the channel power gain is represented as $h_{m,k}^{\left(p,s\right)}$ = $k_{0}{\left(d_{m,k}^{\left(p,s\right)}\right)}^{-\gamma }g_{m,k}^{\left(p,s\right)}s_{m,k}^{\left(p,s\right)}$, where $k_{0}$ denotes the average channel gain at a reference distance $d_{0}=1\;$m and this gain depends on carrier frequency, transmitter and receiver antenna gains and propagation characteristics, γ>0 is the path-loss exponent, $g_{m,k}^{\left(p,s\right)}$ is a Rayleigh distributed r.v., and $s_{m,k}^{\left(p,s\right)}$ is a log-normal r.v. with shadowing parameter $\sigma_{s}$. The same channel model is considered for the other links $h_{m}^{\left(p,d\right)},h_{m,k}^{\left(p,r\right)},h_{m,k}^{\left(s\right)}$, representing respectively the channel gains of the links between the *m*th PU and PBS in direct transmission mode, the *k*th SU and PBS in relay mode, and the *k*th SU when transmitting its own data to the SBS on the fraction of bandwidth leased by the *m*th PU.

In the case of multiple relay SUs being available, the optimal relay selection scheme should be designed. For each PU-SU pair, the transmit power of the PU, $P_{m,k}^{\left(p,s\right)}$ and the transmit power of the relay SU, consumed during relay $P_{m,k}^{\left(p,r\right)}$ and during its own data transmission $P_{m,k}^{\left(s\right)}$, should be designed for performance optimization of the network.

## 3. Proposed Joint Resource Allocation and Relay Selection Scheme

In the following subsections, we formulate an optimization problem, and we present a new solution to the energy efficiency based resource allocation, power allocation, and relay selection.

### 3.1. Energy Efficiency of PUs

The energy efficiency of all the PUs can be expressed as

(1) $$\eta^{\left(p\right)}=\sum_{m=1}^{M}\eta_{m}^{\left(p\right)},$$

where $\eta_{m}^{\left(p\right)}$ denotes the energy efficiency of the *m*th PU. As PUs may choose direct transmission or cooperative transmission through a relay SU for information transmission to the PBS, the energy efficiency of the *m*th PU can be calculated as

(2) $$\eta_{m}^{\left(p\right)}=\beta_{m}^{\left(d\right)}\eta_{m}^{\left(p,d\right)}+\sum_{k=1}^{K}\beta_{m,k}^{\left(c\right)}\eta_{m,k}^{\left(p,c\right)},$$

where $\eta_{m}^{\left(p,d\right)}$ is the energy efficiency of the *m*th PU when using direct mode, while $\beta_{m}^{\left(d\right)}\in \left\{0,1\right\}$ denotes the transmission indicator of the *m*th PU for the direct mode. More specifically, if $\beta_{m}^{\left(d\right)}=1$, the *m*th PU uses direct transmission mode, while for $\beta_{m}^{\left(d\right)}=0$, the PU exploits SU relay assisted transmission. The term $\eta_{m,k}^{\left(p,c\right)}$ denotes the energy efficiency of the *m*th PU when using the *k*th SU as relay node for cooperative transmission mode, while $\beta_{m,k}^{\left(c\right)}\in \left\{0,1\right\}$ is the corresponding transmission and relay selection variable, that is, $\beta_{m,k}^{\left(c\right)}=1$ indicates that the *m*th PU uses the *k*th SU as relay node for cooperative transmission, and $\beta_{m,k}^{\left(c\right)}=0$, otherwise. Note that $\beta_{m}^{\left(d\right)}$ and $\beta_{m,k}^{\left(c\right)}$ are mutually exclusive. In the following subsections, we derive the expression of $\eta_{m}^{\left(p,d\right)}$ and $\eta_{m,k}^{\left(p,c\right)}$, respectively.

#### 3.1.1. Direct Mode

The energy efficiency of the *m*th PU in direct mode, that is, $\eta_{m}^{\left(p,d\right)}$ can be calculated as

(3) $$\eta_{m}^{\left(p,d\right)}=\frac{R_{m}^{\left(p,d\right)}}{P_{m}^{\left(p,d\right)}+P_{c}^{\left(p\right)}},$$

where $P_{m}^{\left(p,d\right)}$ is the power consumed by the *m*th PU when transmitting in direct mode, $P_{c}^{\left(p\right)}$ denotes the circuit power consumption of the *m*th PU, which is assumed to be a constant for all the PUs in this paper, and $R_{m}^{\left(p,d\right)}$ denotes the data rate achieved by the *m*th PU in direct mode, which can be expressed as

(4) $$R_{m}^{\left(p,d\right)}=B\log_{2}\left(1+\frac{P_{m}^{\left(p,d\right)}h_{m}^{\left(p,d\right)}}{\sigma^{2}}\right),$$

where $\sigma^{2}$ is the noise power of the link between the *m*th PU and the PBS. Without loss of generality, the noise power of all the transmission links are assumed to be the same in this paper.

#### 3.1.2. Relay Mode

The energy efficiency of the *m*th PU when using the *k*th SU as relay node for cooperative transmission, denoted by $\eta_{m,k}^{\left(p,c\right)}$, can be expressed as

(5) $$\eta_{m,k}^{\left(p,c\right)}=\frac{R_{m,k}^{\left(p,c\right)}}{P_{m,k}^{\left(p,c\right)}}.$$

The denominator $P_{m,k}^{\left(p,c\right)}$ is the power consumption of the *m*th PU when using the *k*th SU as a relay node and can be calculated as

(6) $$P_{m,k}^{\left(p,c\right)}=P_{m,k}^{\left(p,s\right)}+P_{m,k}^{\left(p,r\right)}+P_{c}^{\left(p\right)}+P_{c}^{\left(s\right)},$$

where $P_{c}^{\left(s\right)}$ denotes the circuit power consumption of the SU which is assumed to be a constant for all the SUs. The numerator $R_{m,k}^{\left(p,c\right)}$ represents the data rate of the *m*th PU when using the *k*th SU as relay node for cooperative transmission. $R_{m,k}^{\left(p,c\right)}$ can be expressed as

(7) $$R_{m,k}^{\left(p,c\right)}=\min\left(R_{m,k}^{\left(p,s\right)},R_{m,k}^{\left(p,r\right)}\right),$$

where $R_{m,k}^{\left(p,s\right)}$ and $R_{m,k}^{\left(p,r\right)}$ are, respectively, the data rate of the link from the *m*th PU to the *k*th SU

(8) $$R_{m,k}^{\left(p,s\right)}=t_{1}\rho_{m}B\log_{2}\left(1+\frac{P_{m,k}^{\left(p,s\right)}h_{m,k}^{\left(p,s\right)}}{\sigma^{2}}\right)$$

and that from the *k*th SU to the PBS when the *k*th SU is chosen as the relay node of the *m*th PU

(9) $$R_{m,k}^{\left(p,r\right)}=\left(1-t_{1}\right)\rho_{m}B\log_{2}\left(1+\frac{P_{m,k}^{\left(p,r\right)}h_{m,k}^{\left(p,r\right)}}{\sigma^{2}}\right).$$

### 3.2. Energy Efficiency of SUs

The energy efficiency of all the SUs in the CCRN, denoted by $\eta^{\left(s\right)}$, can be calculated as

(10) $$\eta^{\left(s\right)}=\sum_{m=1}^{M}\sum_{k=1}^{K}\beta_{m,k}^{\left(c\right)}\eta_{m,k}^{\left(s\right)},$$

where $\eta_{m,k}^{\left(s\right)}$ is the energy efficiency of the *k*th SU when transmitting its own data on the subchannel leased by the *m*th PU, which can be calculated as

(11) $$\eta_{m,k}^{\left(s\right)}=\frac{R_{m,k}^{\left(s\right)}}{P_{m,k}^{\left(s\right)}+P_{c}^{\left(s\right)}},$$

where $R_{m,k}^{\left(s\right)}$ denote the data rate of the *k*th SU when transmitting its own data on the subchannel leased by the *m*th PU

(12) $$R_{m,k}^{\left(s\right)}=\left(1-\rho_{m}\right)B\log_{2}\left(1+\frac{P_{m,k}^{\left(s\right)}h_{m,k}^{\left(s\right)}}{\sigma^{2}}\right).$$

## 4. Energy Efficiency for CCRN Problem Formulation and Solution

The total energy efficiency of the PUs and the SUs can be formulated as:

(13) $$\eta =\eta^{\left(p\right)}+\eta^{\left(s\right)}.$$

In the following, we propose a joint resource allocation, power allocation, and relay selection strategy to maximize the total energy efficiency (13).

### 4.1. Optimization Problem Formulation

The energy efficiency based power allocation, transmission mode and relay selection scheme can be obtained by solving the following optimization problem:

(14) $$\begin{array}{cc} \;\; & \max_{\left[\beta_{m}^{\left(d\right)},\beta_{m,k}^{\left(c\right)},P_{m}^{\left(p,d\right)},P_{m,k}^{\left(p,s\right)},P_{m,k}^{\left(p,r\right)},P_{m,k}^{\left(s\right)}\right]}\eta \\ s.\;t.\;\; & C1:\;\beta_{m}^{\left(d\right)}+\sum_{k=1}^{K}\beta_{m,k}^{\left(c\right)}\leq 1,\;\;m=1,2,...,M\\ \; & C2:\;\sum_{m=1}^{M}\beta_{m,k}^{\left(c\right)}\leq 1,\\ \; & C3:\;P_{m}^{\left(p,d\right)}\leq P_{m}^{\left(p,\max\right)},\mathrm{if}\;\;\beta_{m}^{\left(d\right)}=1,\\ \; & C4:\;P_{m,k}^{\left(p,s\right)}\leq P_{m}^{\left(p,\max\right)},\mathrm{if}\;\;\beta_{m,k}^{\left(c\right)}=1,\\ \; & C5:\;P_{m,k}^{\left(p,r\right)}+P_{m,k}^{\left(s\right)}\leq P_{k}^{\left(s,\max\right)},\mathrm{if}\;\;\beta_{m,k}^{\left(c\right)}=1,\\ \; & C6:\;R_{m}^{\left(p,d\right)}\geq R_{m}^{\left(p,\min\right)},\;\;\mathrm{if}\;\;\beta_{m}^{\left(d\right)}=1,\\ \; & C7:\;R_{m,k}^{\left(p,s\right)}\geq R_{m}^{\left(p,\min\right)},\;\;\mathrm{if}\;\;\beta_{m,k}^{\left(c\right)}=1,\\ \; & C8:\;R_{m,k}^{\left(p,r\right)}\geq R_{m}^{\left(p,\min\right)},\;\;\mathrm{if}\;\;\beta_{m,k}^{\left(c\right)}=1,\\ \; & C9:\;R_{m,k}^{\left(s\right)}\geq R_{m,k}^{\left(s,\min\right)},\;\;\mathrm{if}\;\;\beta_{m,k}^{\left(c\right)}=1. \end{array}$$

C1 represents the transmission mode indicator constraint since it is assumed that every PU can only choose direct transmission mode or relay transmission mode. Similarly, C2 specifies the relay mode indicator constraint as it is assumed that every PU can choose only one SU as its relay node and each SU can only forward packets for one PU. C3, C4 and C5 denote the maximum power constraint where $P_{m}^{\left(p,\max\right)}$ and $P_{k}^{\left(s,\max\right)}$ denote respectively the maximum transmit power of the *m*th PU and the *k*th SU. C6-C9 represent the minimum data rate constraints where $R_{m}^{\left(p,\min\right)}$ and $R_{k}^{\left(s,\min\right)}$ denote respectively the minimum required data rate of the *m*th PU and the *k*th SU.

The optimization problem in (14) can be classified as a nonlinear binary fractional program and therefore difficult to solve using traditional optimization methods [31]. From the optimization constraints C3–C9 given in (14), it can be shown that such an optimization problem can be transformed equivalently into two subproblems: (1) power allocation, (2) transmission mode and relay selection. The solution for each subproblem is presented in the following subsections.

### 4.2. Power Allocation Subproblem

Assuming that the *m*th PU selects direct transmission mode, that is, $\beta_{m}^{\left(d\right)}=1$, the energy efficient optimal power allocation problem can be formulated as:

(15) $$\begin{array}{cc} \;\; & \max_{\left[P_{m}^{\left(p,d\right)}\right]}\frac{R_{m}^{\left(p,d\right)}}{P_{m}^{\left(p,d\right)}+P_{c}^{\left(p\right)}}\\ s.\;t.\;\; & C1:\;P_{m}^{\left(p,d\right)}\leq P_{m}^{\left(p,\max\right)},\\ \; & C2:\;R_{m}^{\left(p,d\right)}\geq R_{m}^{\left(p,\min\right)}. \end{array}$$

For the relay transmission mode, when the *m*th PU selects the *k*th SU as its relay node, that is, $\beta_{m,k}^{\left(c\right)}=1$, the energy efficiency of the PU-SU pair can be expressed as

(16) $$\eta_{m,k}^{\left(p,c\right)}+\eta_{m,k}^{\left(s\right)}.$$

Therefore, the optimal power allocation problem for the *m*th PU and the *k*th SU can be formulated as:

(17) $$\begin{array}{cc} \;\; & \max_{\left[P_{m,k}^{\left(p,s\right)},P_{m,k}^{\left(p,r\right)},P_{m,k}^{\left(s\right)}\right]}\frac{R_{m,k}^{\left(p,c\right)}}{P_{m,k}^{\left(p,c\right)}}+\frac{R_{m,k}^{\left(s\right)}}{P_{m,k}^{\left(s\right)}+P_{c}^{\left(s\right)}}\\ s.\;t.\;\; & C1:\;P_{m,k}^{\left(p,s\right)}\leq P_{m}^{\left(p,\max\right)},\\ \; & C2:\;P_{m,k}^{\left(p,r\right)}+P_{m,k}^{\left(s\right)}\leq P_{k}^{\left(s,\max\right)},\\ \; & C3:\;R_{m,k}^{\left(p,s\right)}\geq R_{m}^{\left(p,\min\right)},\\ \; & C4:\;R_{m,k}^{\left(p,r\right)}\geq R_{m}^{\left(p,\min\right)},\\ \; & C5:\;R_{m,k}^{\left(s\right)}\geq R_{m,k}^{\left(s,\min\right)}. \end{array}$$

#### 4.2.1. Equivalent Problem Transformation

In order to solve the optimization problem in (17), we exploit the fractional programming approach [37]. As we mentioned earlier, we are considering DF at the relay node. In case $R_{m,k}^{\left(p,s\right)}<R_{m,k}^{\left(p,r\right)}$, our optimization problem becomes:

(18) $$\begin{array}{cc} \;\; & \max_{\left[P_{m,k}^{\left(p,s\right)},P_{m,k}^{\left(p,r\right)},P_{m,k}^{\left(s\right)}\right]}\frac{R_{m,k}^{\left(p,s\right)}}{P_{m,k}^{\left(p,c\right)}}+\frac{R_{m,k}^{\left(s\right)}}{P_{m,k}^{\left(s\right)}+P_{c}^{\left(s\right)}}\\ s.\;t.\;\; & C1:\;P_{m,k}^{\left(p,s\right)}\leq P_{m}^{\left(p,\max\right)},\\ \; & C2:\;P_{m,k}^{\left(p,r\right)}+P_{m,k}^{\left(s\right)}\leq P_{k}^{\left(s,\max\right)},\\ \; & C3:\;R_{m,k}^{\left(p,s\right)}<R_{m,k}^{\left(p,r\right)},\\ \; & C4:\;R_{m,k}^{\left(p,s\right)}\geq R_{m}^{\left(p,\min\right)},\\ \; & C5:\;R_{m,k}^{\left(p,r\right)}\geq R_{m}^{\left(p,\min\right)},\\ \; & C6:\;R_{m,k}^{\left(s\right)}\geq R_{m,k}^{\left(s,\min\right)}. \end{array}$$

To proceed, we rewrite the problem in (18) into an equivalent form [38]:

(19) $$\begin{array}{cc} \;\; & \max_{\left[P_{m,k}^{\left(p,s\right)},P_{m,k}^{\left(p,r\right)},P_{m,k}^{\left(s\right)},\alpha_{1},\alpha_{2}\right]}\alpha_{1}+\alpha_{2}\\ s.\;t.\;\; & C1-C6\;\;\mathrm{in}\;\;(18),\\ \; & C7:\;\frac{R_{m,k}^{\left(p,s\right)}}{P_{m,k}^{\left(p,c\right)}}\geq \alpha_{1},\\ \; & C8:\;\frac{R_{m,k}^{\left(s\right)}}{P_{m,k}^{\left(s\right)}+P_{c}^{\left(s\right)}}\geq \alpha_{2}, \end{array}$$

where C7 and C8 denote the energy efficiency constraints.

**Theorem** **1.** If the set ($P_{m,k}^{(p,s)*},P_{m,k}^{(p,r)*},P_{m,k}^{(s)*},\alpha_{1}^{*},\alpha_{2}^{*}$) is the solution for (19), then there exist ν and κ such that ($P_{m,k}^{(p,s)*},P_{m,k}^{(p,r)*},P_{m,k}^{(s)*}$) satisfies that Karush-Kuhn-Tucker (KKT) conditions for $\nu =\bar{\nu }$, $\kappa =\bar{\kappa }$, $\alpha_{1}=\alpha_{1}^{*}$, $\alpha_{2}=\alpha_{2}^{*}$ of the following problem [39]

(20) $$\begin{array}{cc} \;\; & \max_{\left[P_{m,k}^{\left(p,s\right)},P_{m,k}^{\left(p,r\right)},P_{m,k}^{\left(s\right)}\right]}\phi \\ s.\;t.\;\; & C1-C6\;\;\mathrm{in}\;\;(18), \end{array}$$

where $\phi =\nu \left(R_{m,k}^{\left(p,s\right)}-\alpha_{1}P_{m,k}^{\left(p,c\right)}\right)+\kappa \left(R_{m,k}^{\left(s\right)}-\alpha_{2}\left(P_{m,k}^{\left(s\right)}+P_{c}^{\left(s\right)}\right)\right)$. Also the set of solutions satisfies a system of equations for $\nu =\bar{\nu }$, $\kappa =\bar{\kappa }$, $\alpha_{1}=\alpha_{1}^{*}$, and $\alpha_{2}=\alpha_{2}^{*}$, with

(21) $$\begin{array}{c} \left\{\begin{array}{cc} \bar{\nu }=\frac{1}{P_{m,k}^{\left(p,c\right)}} & \bar{\kappa }=\frac{1}{P_{m,k}^{\left(s\right)}+P_{c}^{\left(s\right)}}\\ \alpha_{1}^{*}=\frac{R_{m,k}^{\left(p,s\right)}}{P_{m,k}^{\left(p,c\right)}} & \alpha_{2}^{*}=\frac{R_{m,k}^{\left(s\right)}}{P_{m,k}^{\left(s\right)}+P_{c}^{\left(s\right)}}. \end{array}\right. \end{array}$$

*Proof for Theorem 1 is provided in Appendix A.*

In Theorem 1 we proved that by satisfying the system of equations in (21) among the solutions of (20), the optimization problem in (18) can be solved. It shows as well that for an optimization problem with an objective function in fractional form, there exists an equivalent in subtractive form, that is, ϕ in our case. As a result, we use the equivalent objective function for the rest of the paper.

#### 4.2.2. Energy Efficiency Maximization

In this section we present an iterative algorithm in order to find solutions for the problem in (20). After transforming our problem (18) into (20), it can be easily proven that the tranformed problem is convex and can be solved using the Lagrange dual method [40,41]. Thus, by relaxing the constraints the Lagrange function can be represented as

(22) $$\begin{array}{cc} \; & L(P_{m,k}^{\left(p,r\right)},P_{m,k}^{\left(p,s\right)},P_{m,k}^{\left(s\right)},\lambda ,\delta ,\epsilon ,\xi ,\theta ,\mu ,\nu ,\kappa )=\\ \; & +\nu \left(R_{m,k}^{\left(p,s\right)}-\alpha_{1}\left(P_{m,k}^{\left(p,c\right)}\right)\right)+\kappa \left(R_{m,k}^{\left(s\right)}-\alpha_{2}\left(P_{m,k}^{\left(s\right)}+P_{c}^{\left(s\right)}\right)\right)\\ \; & +\lambda \left(P_{m}^{\left((p,\max\right)}-P_{m,k}^{\left(p,s\right)}\right)+\delta \left(P_{k}^{\left(s,\max\right)}-P_{m,k}^{\left(p,r\right)}-P_{m,k}^{\left(s\right)}\right)\\ & +\epsilon \left(R_{m,k}^{\left(p,r\right)}-R_{m,k}^{\left(p,s\right)}\right)+\xi \left(R_{m,k}^{\left(p,s\right)}-R_{m}^{\left(p,\min\right)}\right)\\ \; & +\delta \left(R_{m,k}^{\left(p,r\right)}-R_{m}^{\left(p,\min\right)}\right)+\mu \left(R_{m,k}^{\left(s\right)}-R_{k}^{\left(s,\min\right)}\right), \end{array}$$

where λ,δ>0 and ϵ,ξ,θ,μ>0 represent the Lagrange multipliers associated with power constraints and minimum data rate requirements, respectively. The corresponding Lagrange dual method can be expressed as follows:

(23) $$\begin{array}{cc} \;\; & \min_{\left[\lambda ,\delta ,\epsilon ,\xi ,\theta ,\mu \right]}\;\;\max_{\left[P_{m,k}^{\left(p,r\right)},P_{m,k}^{\left(p,s\right)},P_{m,k}^{\left(s\right)}\right]}L\\ s.\;t.\;\; & \lambda \geq 0,\delta \geq 0,\epsilon \geq 0,\xi \geq 0,\theta \geq 0,\mu \geq 0. \end{array}$$

The above dual problem can be solved by optimizing the transmit power for a fixed set of Lagrange multipliers, and updating the Lagrange multipliers iteratively as well as our parameters $\nu ,\kappa ,\alpha_{1},\alpha_{2}$. For a given set of Lagrange multipliers λ,δ,ϵ,ξ,θ,μ, the locally optimal transmit powers can be obtained through calculating the derivative of $L(P_{m,k}^{\left(p,r\right)},P_{m,k}^{\left(p,s\right)},P_{m,k}^{\left(s\right)},\lambda ,\delta ,\epsilon ,\xi ,\theta ,\mu )$ over the transmit powers and setting it to zero thus obtaining

(24) $$P_{m,k}^{(p,s)*}={\left[\frac{t_{1}\rho_{m}B\left(\nu +\xi -\epsilon \right)}{(\lambda +\nu \alpha_{1})ln2}-\frac{\sigma^{2}}{h_{m,k}^{\left(p,s\right)}}\right]}^{+}$$

(25) $$P_{m,k}^{(p,r)*}={\left[\frac{\left(1-t_{1}\right)\rho_{m}B\left(\epsilon +\theta \right)}{(\delta +\nu \alpha_{1})ln2}-\frac{\sigma^{2}}{h_{m,k}^{\left(p,r\right)}}\right]}^{+}$$

(26) $$P_{m,k}^{(s)*}={\left[\frac{\left(1-\rho_{m}\right)B\left(\mu +\kappa \right)}{(\delta +\kappa \alpha_{2})ln2}-\frac{\sigma^{2}}{h_{m,k}^{\left(s\right)}}\right]}^{+},$$

where ${\left[z\right]}^{+}=\max\left(0,z\right)$.

In case $R_{m,k}^{\left(p,s\right)}\geq R_{m,k}^{\left(p,r\right)}$, a similar approach can be followed, leading to the optimization problem:

(27) $$\begin{array}{cc} \;\; & \max_{\left[P_{m,k}^{\left(p,s\right)},P_{m,k}^{\left(p,r\right)},P_{m,k}^{\left(s\right)}\right]}\frac{R_{m,k}^{\left(p,s\right)}}{P_{m,k}^{\left(p,c\right)}}+\frac{R_{m,k}^{\left(s\right)}}{P_{m,k}^{\left(s\right)}+P_{c}^{\left(s\right)}}\\ s.\;t.\;\; & C1:\;P_{m,k}^{\left(p,s\right)}\leq P_{m}^{\left(p,\max\right)},\\ \; & C2:\;P_{m,k}^{\left(p,r\right)}+P_{m,k}^{\left(s\right)}\leq P_{k}^{\left(s,\max\right)},\\ \; & C3:\;R_{m,k}^{\left(p,s\right)}\geq R_{m,k}^{\left(p,r\right)},\\ \; & C4:\;R_{m,k}^{\left(p,s\right)}\geq R_{m}^{\left(p,\min\right)},\\ \; & C5:\;R_{m,k}^{\left(p,r\right)}\geq R_{m}^{\left(p,\min\right)},\\ \; & C6:\;R_{m,k}^{\left(s\right)}\geq R_{m,k}^{\left(s,\min\right)}. \end{array}$$

By applying to (28) the same transformation explained in Theorem 1 and applying Lagrange dual method to the transformed problem, we are able to calculate the transmit powers:

(28) $$P_{m,k}^{(p,s)*}={\left[\frac{t_{1}\rho_{m}B\left(\xi +\epsilon \right)}{(\lambda +\nu \alpha_{1})ln2}-\frac{\sigma^{2}}{h_{m,k}^{\left(p,s\right)}}\right]}^{+}$$

(29) $$P_{m,k}^{(p,r)*}={\left[\frac{\left(1-t_{1}\right)\rho_{m}B\left(\nu -\epsilon +\theta \right)}{(\delta +\nu \alpha_{1})ln2}-\frac{\sigma^{2}}{h_{m,k}^{\left(p,r\right)}}\right]}^{+}$$

(30) $$P_{m,k}^{(s)*}={\left[\frac{\left(1-\rho_{m}\right)B\left(\mu +\kappa \right)}{(\delta +\kappa \alpha_{2})ln2}-\frac{\sigma^{2}}{h_{m,k}^{\left(s\right)}}\right]}^{+}.$$

Based on these solutions, we propose a two-layer optimization algorithm. In the inner layer the Lagrange multipliers in (24)–(26) and (28)–(30) can be updated through using the subgradient method [42], that is,

(31) $$\lambda \left(n+1\right)={\left[\lambda \left(n\right)-\psi \left(P_{m}^{\left(p,\max\right)}-P_{m,k}^{\left(p,s\right)}\right)\right]}^{+}$$

(32) $$\delta \left(n+1\right)={\left[\delta \left(n\right)-\psi \left(P_{k}^{\left(s,\max\right)}-P_{m,k}^{\left(p,r\right)}-P_{m,k}^{\left(s\right)}\right)\right]}^{+}$$

(33) $$\epsilon \left(n+1\right)={\left[\epsilon \left(n\right)-\psi |R_{m,k}^{\left(p,r\right)}-R_{m,k}^{\left(p,s\right)}|\right]}^{+}$$

(34) $$\xi \left(n+1\right)={\left[\xi \left(n\right)-\psi \left(R_{m,k}^{\left(p,s\right)}-R_{m}^{\left(p,\min\right)}\right)\right]}^{+}$$

(35) $$\theta \left(n+1\right)={\left[\theta \left(n\right)-\psi \left(R_{m,k}^{\left(p,r\right)}-R_{m}^{\left(p,\min\right)}\right)\right]}^{+}$$

(36) $$\mu \left(n+1\right)={\left[\mu \left(n\right)-\psi \left(R_{m,k}^{\left(s\right)}-R_{k}^{\left(s,\min\right)}\right)\right]}^{+},$$

where ψ denotes the learning rate.

In the outer layer the parameters $\nu ,\kappa ,\alpha_{1},\alpha_{2}$ can be updated using Newton-Raphson method [43], that is,

(37) $$\nu \left(n+1\right)=\nu \left(n\right)-\chi \left(\frac{\nu \left(n\right)P_{m,k}^{\left(p,c\right)}-1}{P_{m,k}^{\left(p,c\right)}}\right)$$

(38) $$\kappa \left(n+1\right)=\kappa \left(n\right)-\chi \left(\frac{\kappa (P_{m,k}^{\left(s\right)}+P_{c}^{\left(s\right)})-1}{P_{m,k}^{\left(s\right)}+P_{c}^{\left(s\right)}}\right)$$

(39) $$\alpha_{1}\left(n+1\right)=\alpha_{1}\left(n\right)-\chi \left(\frac{\alpha_{1}\left(n\right)P_{m,k}^{\left(p,c\right)}-\tau }{P_{m,k}^{\left(p,c\right)}}\right)$$

(40) $$\alpha_{2}\left(n+1\right)=\alpha_{2}\left(n\right)-\chi \left(\frac{\alpha_{2}\left(n\right)\left(P_{m,k}^{\left(s\right)}+P_{c}^{\left(s\right)}\right)-R_{m,k}^{\left(s\right)}}{P_{m,k}^{\left(s\right)}+P_{c}^{\left(s\right)}}\right),$$

where χ denotes the learning rate and $\tau =\min(R_{m,k}^{\left(p,s\right)},R_{m,k}^{\left(p,r\right)})$. Detailed steps of our algorithm are shown in Algorithm 1.
**Algorithm 1.** Power Allocation Optimization1:Set tollerance ζ and maximum number of iterations $n_{\max}$
Let n=0, choose ψ∈(0,1), χ∈(0,1)
Choose arbitrarily $P_{m,k}^{\left(p,s\right)}\left(0\right)$, $P_{m,k}^{\left(p,r\right)}\left(0\right)$,$P_{m,k}^{\left(s\right)}\left(0\right)$ such that satisfies
power constraints
Calculate $R_{m,k}^{\left(p,s\right)}\left(0\right)$, $R_{m,k}^{\left(p,r\right)}\left(0\right)$ and $R_{m,k}^{\left(s\right)}\left(0\right)$
**if**$R_{m,k}^{\left(p,s\right)}<R_{m,k}^{\left(p,r\right)}$

**then**$\tau =R_{m,k}^{\left((p,s\right)}$
**else**$\tau =R_{m,k}^{\left(p,r\right)}$
**end**
Let



$\left\{\begin{array}{c} \nu \left(n\right)=\frac{1}{P_{m,k}^{\left(p,c\right)}\left(n\right)}\\ \kappa \left(n\right)=\frac{1}{P_{m,k}^{\left(s\right)}\left(n\right)+P_{c}^{\left(s\right)}}\\ \alpha_{1}\left(n\right)=\frac{\tau }{P_{m,k}^{\left(p,c\right)}\left(n\right)}\\ \alpha_{2}\left(n\right)=\frac{R_{m,k}^{\left(s\right)}\left(n\right)}{P_{m,k}^{\left(s\right)}\left(n\right)+P_{c}^{\left(s\right)}} \end{array}\right.$
Initialize the Lagrange multipliers λ,δ,ϵ,ξ,θ,μ2:**if**$\tau =R_{m,k}^{\left(p,s\right)}$

update $P_{m,k}^{\left(p,s\right)}$ and $P_{m,k}^{\left(p,r\right)}$ with (24) and (25) and

obtain $P_{m,k}^{(p,s)*}$ and $P_{m,k}^{(p,r)*}$
**else**

update $P_{m,k}^{\left(p,s\right)}$ and $P_{m,k}^{\left(p,r\right)}$ with (28) and (29) and

obtain $P_{m,k}^{(p,s)*}$ and $P_{m,k}^{(p,r)*}$
**end**
Update $P_{m,k}^{\left(s\right)}$ with (26) and obtain $P_{m,k}^{(s)*}$3:Update the Lagrange multipliers using (31)–(36)4:Compute $\phi^{*}$ with $P_{m,k}^{(p,s)*}$, $P_{m,k}^{(p,r)*}$ and $P_{m,k}^{(s)*}$
**if**$|\phi^{*}-\phi^{\left(n\right)}|\leq \zeta$

$P_{m,k}^{\left(p,s\right)}\left(n+1\right)=P_{m,k}^{(p,s)*}$, $P_{m,k}^{\left(p,r\right)}\left(n+1\right)=P_{m,k}^{(p,r)*}$, $P_{m,k}^{\left(s\right)}\left(n+1\right)=P_{m,k}^{(s)*}$

**exit program** (convergence reached)
**else**

$P_{m,k}^{\left(p,s\right)}\left(n\right)=P_{m,k}^{(p,s)*}$, $P_{m,k}^{\left(p,r\right)}\left(n\right)=P_{m,k}^{(p,r)*}$, $P_{m,k}^{\left(s\right)}\left(n\right)=P_{m,k}^{(s)*}$

ϕ(n) = $\phi^{*}$

update $\nu ,\kappa ,\alpha_{1},\alpha_{2}$ using (37)–(40)

Update $R_{m,k}^{\left(p,s\right)}\left(n+1\right),R_{m,k}^{\left(p,r\right)}\left(n+1\right),R_{m,k}^{\left(s\right)}\left(n+1\right)$

n←n+1
**end**
**go to** step 2 unless *n* reached $n_{\max}$

For the problem in (15), a similar approach can be followed. By transforming it using Theorem 1 and applying the Lagrange dual method, the optimal transmit power for the direct mode can be obtained.

### 4.3. Transmission Mode and Relay Selection Subproblem

Given the optimal transmit power of the PUs and the SUs, the total energy efficiency of the network can be calculated as:

(41) $$\begin{array}{c} \eta^{*}=\sum_{m=1}^{M}\sum_{k=1}^{K}\left(\beta_{m}^{\left(d\right)}\eta_{m}^{*}+\beta_{m,k}^{\left(c\right)}\eta_{m,k}^{*}\right). \end{array}$$

The transmission mode and relay selection subproblem can be formulated as:

(42) $$\begin{array}{cc} \;\; & \max_{\left[\beta_{m}^{\left(d\right)},\beta_{m,k}^{\left(c\right)}\right]}\eta^{*}\\ s.\;t.\;\; & C1:\;\beta_{m}^{\left(d\right)}+\sum_{k=1}^{K}\beta_{m,k}^{\left(c\right)}\leq 1,\\ \; & C2:\;\sum_{m=1}^{M}\beta_{m,k}^{\left(c\right)}\leq 1, \end{array}$$

which is a linear binary optimization problem, that can be solved using graph-based optimization method [44]. To implement such optimization problem, we construct two tables: Table 1a, whose columns contain the maximum energy efficiency when the *m*th PU chooses direct transmission mode; Table 1b, which contain the optimal energy efficiency when the *m*th PU chooses the *k*th SU as a relay node for cooperation mode.

It can be seen that the optimal transmission mode and relay selection solution of (42) is equivalent to finding the maximum sum of the energy efficiency elements which are chosen from various rows and columns, except for the direct mode. From (42), we can see that when the energy efficiency of the cell where only direct transmission is available is larger than all the energy efficiency of a cell where relay transmission is adopted, the PU should choose the direct mode. Hence, to solve the optimization problem formulated in (42), for the *m*th row, 1≤m≤M, we first compare the element in direct mode with the other elements in the same row number in Table 1b; if the energy efficiency of the PU obtained in direct mode is the largest, then we set $\beta_{m}^{\left(d\right)}=1,\;\beta_{m,k}^{\left(c\right)}=0$, and delete the corresponding row in Table 1b. For the remaining, we can solve the optimal relay selection subproblem for cooperation transmission mode.

Given the constraints on both PUs and SUs, the optimal relay selection subproblem can be described as a bipartite graph, and the problem of finding the optimal relay can be regarded as an optimal matching algorithm in the bipartite graph that can be solved by a modified Kuhn-Munkres (K-M) algorithm [45,46]. A brief description of this algorithm and of its complexity is presented in Appendix B.

Based on the optimal relay selection results, we obtain the corresponding energy efficiency for cooperative mode, which is compared with that obtained from the direct mode for each PU. If the latter is larger, we set in direct mode the corresponding PU and rerun the K-M algorithm until the optimal energy efficiency obtained for cooperative mode is larger than that from the direct mode for all the PUs. In Algorithm 2, we present a brief description of the algorithm.
**Algorithm 2.** Transmission Mode and Relay Selection Optimization
1:Solving optimal power allocation subproblem
to obtain $\eta_{m}^{*}$ and $\eta_{m,k}^{*}$, construct Table 12:**for** the *m*th PU
   **if**
$\eta_{m}^{*}>\eta_{m,k}^{*}$, ∀1≤k≤K

   **then**

      set $\beta_{m}^{\left(p,d\right)}=1$

      delete the *m*th row in Table 1b

**end**
**end**
Table 1b is updated3:**repeat**4:Apply K-M algorithm on Table 1b to find
optimal relay node obtaining $\beta_{m,k}^{\left(c\right)}$5:**if**$\beta_{m,k}^{\left(c\right)}\eta_{m,k}^{*}<\eta_{m}^{*},\;1\leq m\leq M$

      set $\beta_{m}^{\left(p,d\right)}=1$

      set $\beta_{m,k}^{\left(c\right)}=0$

Update Table 1b removing the *m*th row
**end**6:**until**$\beta_{m,k}^{\left(c\right)}\eta_{m,k}^{*}>\eta_{m}^{*}$,∀ PU${}_{m}$ in Table 1b.

## 5. Results

In this section, we evaluate the system performance and effectiveness of the proposed solution throughout Monte Carlo simulations. A single PUs’ cell of 250m radius is considered (see Figure 1), and a summary of the scenario parameters are shown in Table 2. Results are obtained averaging over 5000 independent configuration snapshots, each one with different PUs’ and SUs’ positions within the cell (PUs and SUs are uniformly distributed within the area), and independent realizations of shadowing and fading. Regarding the time slot subdivision between PU and SU, we initially assume $t_{1}=T/2$, which is the choice maximizing energy efficiency, as shown in Section 5.6.

### 5.1. Convergence of the Iterative Algorithm

**Figure 3:**
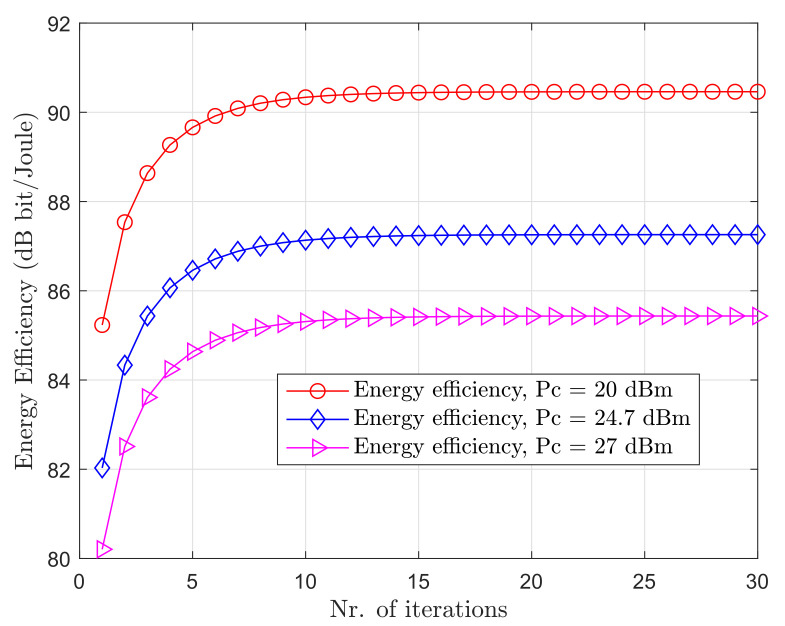
Energy efficiency versus number of iterations with M=10 and K=10, for different circuit power consumption $P_{c}$.

In Figure 3, we show the energy efficiency in deciBel, that is, $\eta \left(\mathrm{dB}\right)=10\log_{10}\eta$, for different values of PUs’ and SUs’ circuit power, $P_{c}$, as a function of the number of iterations, to illustrate the convergence of the proposed algorithm. It can be seen that the iterative algorithm generates a nondecreasing sequence and converges to a stable point within 15 iterations. In addition, we can observe the effect of $P_{c}$ to energy efficiency. As expected, the energy efficiency decreases with the increase of $P_{c}$.

### 5.2. Energy Efficiency Versus Maximum Transmit Power

**Figure 4:**
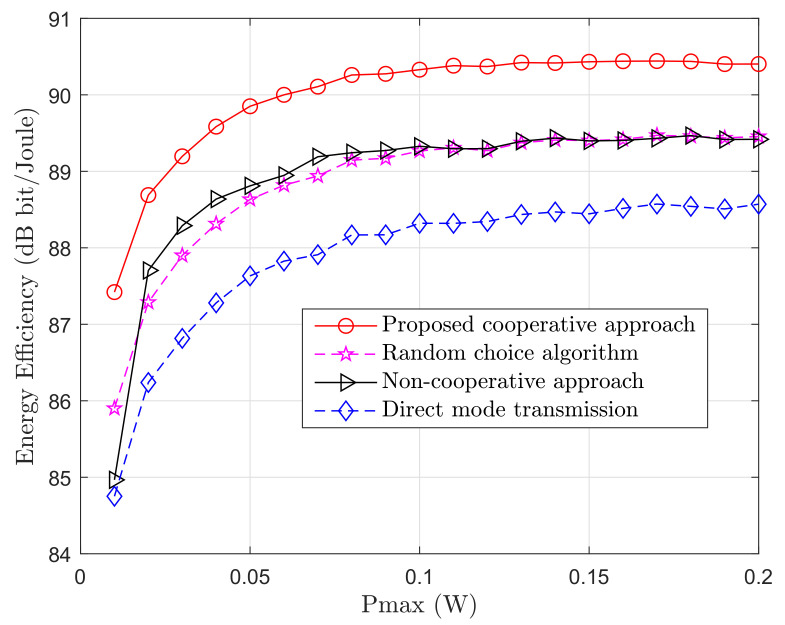
Energy efficiency varying the maximum transmit power. Three different algorithms are compared. *M* and *K* in this case change randomly from 1 to 10.

In Figure 4, the average energy efficiency varying the maximum transmit power, $P_{\max}$, is presented. The number of PUs and SUs is chosen randomly at every adaptation with a maximum number of 10 PUs and 10 SUs. From the figure, it can be observed that the energy efficiency increases with an increase of $P_{\max}$ up to approximately 0.1W, beyond which the energy efficiency saturates. Besides, we compare the performance of our solution with three different algorithms:

- A random choice algorithm in which PUs choose cooperative relays randomly.
- A non-cooperative approach in which a similar system model is considered but PUs choose relays to increase their own energy efficiency, instead of maximizing the total energy efficiency of the system.
- A system in which there are only PUs, and therefore only direct transmission is possible.

As can be observed, the proposed algorithm outperforms the other three solutions. In addition, compared to a system where only PUs are present, we can show that with our approach having the same available spectrum, higher energy efficiency can be achieved, and also more users are able to use the resources available.

### 5.3. Energy Efficiency Versus Number of Pus

**Figure 5:**
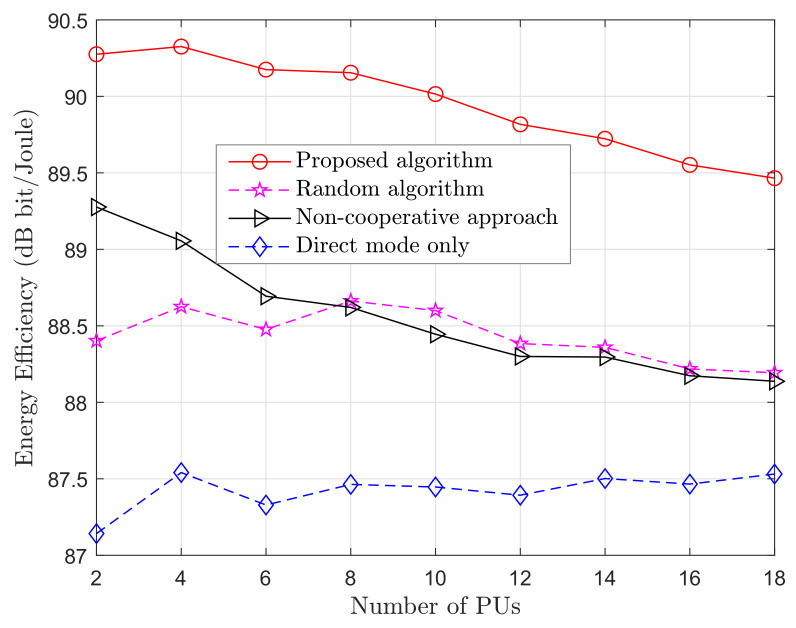
Energy efficiency varying number of PUs. *M* varies from 2 to 18 and K=10.

Figure 5 illustrates the effect that an increasing number of PUs have on energy efficiency when the number of SUs is set to 10. When the number of PUs is smaller than the number of SUs, we can observe a higher energy efficiency achieved. However, increasing the number of PUs, the energy efficiency decreases because of the increased contention of PUs to choose the most convenient cooperative relay. When the number of PUs becomes larger than the number of SUs, the decline of the curve becomes sharper. This comes from the fact that not every PU can choose a cooperative relay and is bounded to perform a direct transmission. As we have shown before, the energy efficiency based only on direct transmission is lower than the one based on the cooperative relay.

### 5.4. Energy Efficiency Versus Path Loss Exponent

**Figure 6:**
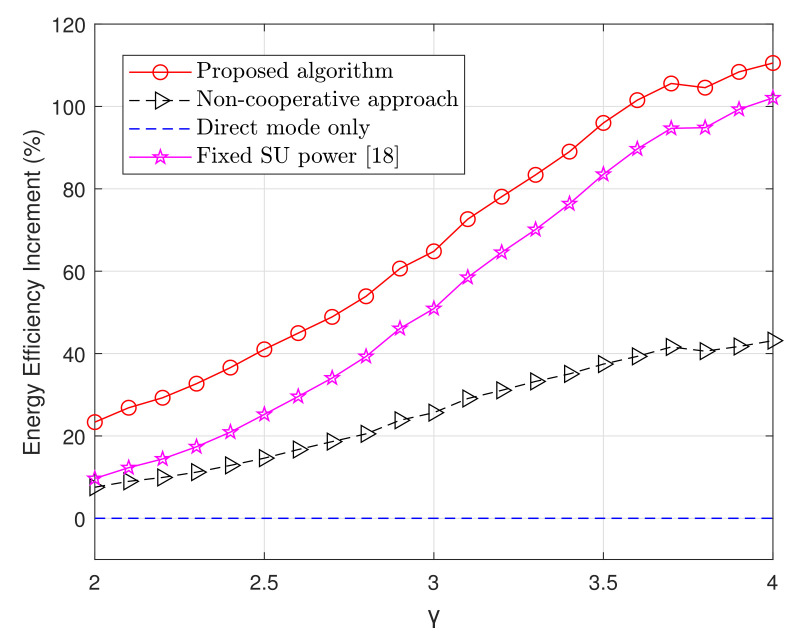
Energy efficiency increment with respect to direct mode varying the path loss exponent. *M* and *K* change randomly from 1 to 15 while $\sigma_{s}$ = 8dB.

In Figure 6, the average energy efficiency varying the path-loss exponent, γ, is presented. With the increase of γ, the energy efficiency decreases; this is due to the increased attenuation, which reflects in a lower data rate. Also, it can be seen that our algorithm outperforms the energy efficiency obtained from the scheme proposed in Reference [18], in which only PUs’ power is optimized, the non-cooperative approach, as well as a system where only PUs are present. In harsh propagation environments with γ>3.5, the energy efficiency of the proposed algorithm outperforms the direct transmission scenario by approximately 110% and the proposed scheme in Reference [18] by 10%.

### 5.5. Energy Efficiency Versus Shadowing Standard Deviation

**Figure 7:**
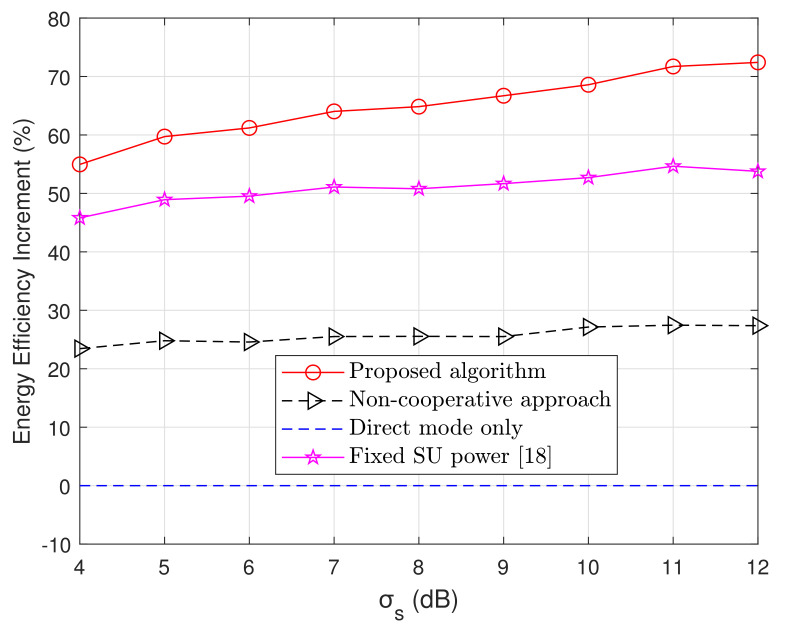
Energy efficiency increment with respect to direct mode versus shadowing standard deviation. *M* and *K* in this case change randomly within 1 and 15 while γ = 3.

In Figure 7, we illustrate the average energy efficiency varying the shadowing standard deviation. As can be seen, also considering the previous figure, the proposed algorithm is more robust to shadowing and attenuation hence offers better performance in harsh propagation environments. The proposed scheme outperforms the direct transmission scenario by 72% and the proposed scheme in Reference [18] by nearly 20%.

### 5.6. Energy Efficiency Versus Time Slot Division

**Figure 8:**
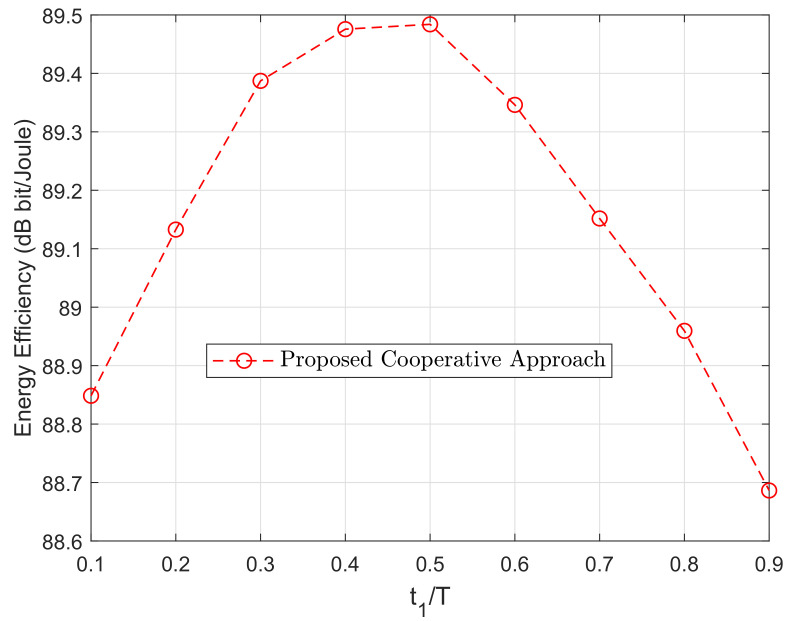
Energy efficiency versus time slot division in the relay transmission link. The parameters *M* and *K* are random variables uniformly distributed from 1 to 10.

In Figure 8, the average energy efficiency varying the time slot division chosen in the two relay transmission links is illustrated. As we notice in the figure, when both links have the same duration, we can achieve the highest value of energy efficiency. In addition, it can be observed that the variation between the maximum, for $t_{1}/T=0.5$, and minimum value, for $t_{1}/T=0.9$, of energy efficiency is not very high. This fact confirms the robustness of the proposed algorithm to the choice of the time slot division in the relay transmission.

## 6. Conclusions

In this paper, a novel energy-efficient architecture for CRN is proposed where each PU can choose one SU as a relay node. To encourage the cooperative behavior of the SUs, PUs lease a fraction of their allocated spectrum to the relay SUs to transmit their data. We proposed a centralized resource management network architecture to achieve a performance enhancement of the network. The resource allocation problem is formulated as a constrained sum energy efficiency maximization problem. Our analysis shows that this problem can be divided into two subproblems: (1) power allocation, (2) transmission mode and relay selection. To tackle the sum-of-ratio form of the power allocation subproblem, we transform it into a subtractive form optimization problem, and then closed-form optimal powers are calculated through a two-layer optimization. A modified K-M algorithm bipartite matching algorithm is used to solve the transmission mode and relay selection subproblem. System simulations performed in a typical multiuser case show that the proposed algorithm converges to the solution within a small number of iterations. In addition, simulations show an increment in the energy efficiency of the PUs network performance compared to previously proposed algorithms when channel conditions get worse. In addition our proposed algorithm shows a robustness to the choice of the time division in the relay transmission. Furthermore, in the system simulations we illustrated the effect that an increasing number of PUs have on the energy efficiency of the cell. Future work will deal with the use of 3D image-based algorithms to acquire more realistic channel characteristics in more realistic scenarios. In addition, we will consider comparing our algorithm with some existing non-CCRNs-based schemes.

## Figures and Tables

**Table 1:** Tables of direct mode and cooperative mode.

(a) Direct Mode		(b) Cooperative Mode			
	**Direct Mode**	**Coop. with SU** ${}_{1}$	**Coop. with SU** ${}_{2}$	⋯	**Coop. with SU** ${}_{K}$
PU${}_{1}$	$\eta_{1}^{*}$	$\eta_{1,1}^{*}$	$\eta_{1,2}^{*}$	⋯	$\eta_{1,K}^{*}$
PU${}_{2}$	$\eta_{2}^{*}$	$\eta_{2,1}^{*}$	$\eta_{2,2}^{*}$	⋯	$\eta_{2,K}^{*}$
⋯		⋯			⋯
PU${}_{M}$	$\eta_{M}^{*}$	$\eta_{M,1}^{*}$	$\eta_{M,2}^{*}$	⋯	$\eta_{M,K}^{*}$

**Table 2:** System parameters.

Cell radius	250m
Small scale fading distribution	Rayleigh fading
Carrier frequency	3.5GHz
Bandwidth	50MHz
Noise power per link, $\sigma^{2}$	−90dBm
Shadowing, $s_{m,k}^{\left(p,s\right)}$	Log-normal with standard deviation of 8dB
Average channel gain at 1 m, $k_{0}$	−39dB
Path-loss exponent, γ	3
Circuit power per UE (PU or SU), $P_{c}$	20dBm
Maximum transmit power per UE, $P_{\max}$	24dBm
Minimum data rate constraint, $R_{m}^{\left(p,\min\right)}$	100Mbit/s
Fraction of bandwidth, $\rho_{m}$	0.66

## Appendix A. Proof of Theorem 1

To solve the problem in (19), Lagrange dual method is used [41]. For this purpose, we first need the Lagrange function of this problem. The Lagrangian can be written as:

(A1) $$\begin{array}{cc} \; & L\left(P_{m,k}^{\left(p,r\right)},P_{m,k}^{\left(p,s\right)},P_{m,k}^{\left(s\right)},\alpha_{1},\alpha_{2},\lambda ,\delta ,\epsilon ,\xi ,\theta ,\mu ,\nu ,\kappa \right)=\alpha_{1}+\alpha_{2}\\ \; & +\lambda \left(P_{m}^{\left(p,\max\right)}-P_{m,k}^{\left(p,s\right)}\right)+\delta \left(P_{k}^{\left(s,\max\right)}-P_{m,k}^{\left(p,r\right)}-P_{m,k}^{\left(s\right)}\right)\\ & +\epsilon \left(R_{m,k}^{\left(p,r\right)}-R_{m,k}^{\left(p,s\right)}\right)+\xi \left(R_{m,k}^{\left(p,s\right)}-R_{m}^{\left(p,\min\right)}\right)\\ \; & +\delta \left(R_{m,k}^{\left(p,r\right)}-R_{m}^{\left(p,\min\right)}\right)+\mu \left(R_{m,k}^{\left(s\right)}-R_{k}^{\left(s,\min\right)}\right)\\ & +\nu \left(R_{m,k}^{\left(p,s\right)}-\alpha_{1}\left(P_{m,k}^{\left(p,c\right)}\right)\right)+\kappa \left(R_{m,k}^{\left(s\right)}-\alpha_{2}\left(P_{m,k}^{\left(s\right)}+P_{c}^{\left(s\right)}\right)\right). \end{array}$$

If the set of solutions ($P_{m,k}^{(p,s)*},P_{m,k}^{(p,r)*},P_{m,k}^{(s)*},\alpha_{1}^{*},\alpha_{2}^{*}$) is the solution of the problem in (19), then exist the Lagrange multipliers $\bar{\lambda },\bar{\delta },\bar{\epsilon },\bar{\xi },\bar{\theta },\bar{\mu },\bar{\nu },\bar{\kappa }$ in order to satisfy the KKT conditions [39] for the problem (19). The conditions are as follows:

(A2) $$\begin{array}{c} \left\{\begin{array}{c} \frac{\partial L}{\partial P_{m,k}^{\left(p,s\right)}}=\bar{\nu }\left(P_{m,k}^{\left(p,s\right)}-\alpha_{1}^{*}\right)-\bar{\lambda }-\bar{\epsilon }R_{m,k}^{\left(p,s\right)}=0\\ \frac{\partial L}{\partial P_{m,k}^{\left(p,r\right)}}=\left(\bar{\epsilon }+\bar{\theta }\right)R_{m,k}^{\left(p,r\right)}-\bar{\nu }\alpha_{1}^{*}-\bar{\delta }=0\\ \frac{\partial L}{\partial P_{m,k}^{\left(s\right)}}=\left(\bar{\mu }+\bar{\kappa }\right)R_{m,k}^{\left(s\right)}-\bar{\kappa }\alpha_{2}^{*}-\bar{\delta }=0 \end{array}\right. \end{array}$$

(A3) $$\begin{array}{cc} \; & \frac{\partial L}{\partial \alpha_{1}}=1-\bar{\nu }P_{m,k}^{\left(p,c\right)}=0 \end{array}$$

(A4) $$\begin{array}{cc} \; & \frac{\partial L}{\partial \alpha_{2}}=1-\bar{\kappa }\left(P_{m,k}^{\left(s\right)}+P_{c}^{\left(s\right)}\right)=0 \end{array}$$

(A5) $$\begin{array}{cc} \; & \frac{\partial L}{\partial \lambda }=P_{m}^{p,max}-P_{m,k}^{\left(p,s\right)}=0 \end{array}$$

(A6) $$\begin{array}{cc} \; & \frac{\partial L}{\partial \delta }=P_{k}^{s,max}-P_{m,k}^{\left(p,r\right)}-P_{m,k}^{\left(s\right)}=0 \end{array}$$

(A7) $$\begin{array}{cc} \; & \frac{\partial L}{\partial \epsilon }=R_{m,k}^{\left(p,r\right)}-R_{m,k}^{\left(p,s\right)}=0 \end{array}$$

(A8) $$\begin{array}{cc} \; & \frac{\partial L}{\partial \xi }=R_{m,k}^{\left(p,s\right)}-R_{m}^{\left(p,\min\right)}=0 \end{array}$$

(A9) $$\begin{array}{cc} \; & \frac{\partial L}{\partial \theta }=R_{m,k}^{\left(p,r\right)}-R_{m}^{\left(p,\min\right)}=0 \end{array}$$

(A10) $$\begin{array}{cc} \; & \frac{\partial L}{\partial \xi }=R_{m,k}^{\left(s\right)}-R_{k}^{\left(s,\min\right)}=0 \end{array}$$

(A11) $$\begin{array}{cc} \; & \frac{\partial L}{\partial \nu }=R_{m,k}^{\left(p,s\right)}-\alpha_{1}^{*}P_{m,k}^{\left(p,c\right)}=0 \end{array}$$

(A12) $$\begin{array}{cc} \; & \frac{\partial L}{\partial \kappa }=R_{m,k}^{\left(s\right)}-\alpha_{2}^{*}\left(P_{m,k}^{\left(p,c\right)}+P_{c}^{\left(s\right)}\right)=0 \end{array}$$

Considering that $P_{m,k}^{\left(p,c\right)}>0$ and $P_{m,k}^{\left(p,c\right)}+P_{c}^{\left(s\right)}>0$ then from (A3) and (A4) we have

(A13) $$\begin{array}{c} \left\{\begin{array}{c} \bar{\nu }=\frac{1}{P_{m,k}^{\left(p,c\right)}}\\ \bar{\kappa }=\frac{1}{P_{m,k}^{\left(s\right)}+P_{c}^{\left(s\right)}} \end{array}\right. \end{array}$$

and (A11) and (A12) are equivalent to

(A14) $$\begin{array}{c} \left\{\begin{array}{c} \alpha_{1}^{*}=\frac{R_{m,k}^{\left(p,s\right)}}{P_{m,k}^{\left(p,c\right)}}\\ \alpha_{2}^{*}=\frac{R_{m,k}^{\left(s\right)}}{P_{m,k}^{\left(s\right)}+P_{c}^{\left(s\right)}}. \end{array}\right. \end{array}$$

The system Equations (A2), (A5)–(A10) are the KKT conditions of the optimization problem in (20) for $\nu =\bar{\nu }$, $\kappa =\bar{\kappa }$, $\alpha_{1}=\alpha_{1}^{*}$, $\alpha_{2}=\alpha_{2}^{*}$.

## Appendix B. Summary of the Kuhn-Munkres Algorithm

For the sake of completeness, we briefly introduce some definitions and a theorem regarding the K-M algorithm.

Bipartite: A bipartite graph is a graph whose vertices can be divided into two disjoint sets *U* and *V*, such that every edge connects a vertex in *U* to one in *V*. It can be represented as G=(U,V,E) with *E* denoting the edges of the graph.

Weighted bipartite: A weighted bipartite is a bipartite in which each edge (u,v) has a weight factor w(u,v).

Matching: A matching in a graph is a subset H⊆G. If *H* and *G* share the same vertex set, then *H* is called a complete matching. The size of a matching is denoted as |H| which equals to the number of edges in *H*.

Feasible vertex labeling: A feasible vertex labeling in *G* is a real-valued function *l* on U∪V such that for u∈U and v∈V,

(A15) l(u)+l(v)≥w(u,v).

**Equality subgraph**: If *l* is a feasible labeling, we denote a subgraph of *G* as $G_{l}$ which contains a number of edges and the endpoints of these edges. If the edges of $G_{l}$ meet the condition l(u)+l(v)≥w(u,v), then $G_{l}$ is called the equality subgraph for *l*.

**Theorem** **A1.**
*If l is a feasible vertex labeling for G, and H is a complete matching of U to V with $H\subseteq G_{l}$, then H is an optimal assignment of U to V.*

### Appendix B.1. Solving Optimal Relay Selection Problem Based on K-M Algorithm

Applying K-M algorithm to solve the optimal relay selection problem of the PUs, a weighted bipartite graph *G* with a bipartite division $G^{0}=\left(U,V,E\right)$ is constructed, where the set of vertices *U* represents the set of PUs, that is, $U=[{\mathrm{PU}}_{1},{\mathrm{PU}}_{2},\ldots ,{\mathrm{PU}}_{M}]$ and the set of vertices *V* represents the set of SUs, $V=[{\mathrm{SU}}_{1},{\mathrm{SU}}_{2},\ldots ,{\mathrm{SU}}_{K}]$. The weight of the edge $\left({\mathrm{PU}}_{m},{\mathrm{SU}}_{k}\right)$ in the weighted bipartite graph can be defined as the joint energy efficiency of the *m*th PU and the *k*th SU, that is, $\eta_{m,k}^{\left(p,c\right)}+\eta_{m,k}^{\left(s\right)}$, m=1,…,M and k=1,…,K.

The steps of solving the optimal relay selection problem based on K-M algorithm can be described as follows:

1. Find initial feasible vertex labeling and determine $G_{l}^{0}$ and choose an arbitrary matching *H* in $G_{l}^{0}$.
2. If *H* is a maximum matching for *G*, then the optimization problem is solved. Otherwise, the label having not being allotted by the distribution *H* is selected in $G_{l}^{0}$. Set S=U, and T=Φ, which denotes the empty set.
3. $N_{G_{l}^{0}}\left(S\right)$ denotes the collection of points which connect with *S* in $G_{l}^{0}$. If $N_{G_{l}^{0}}\left(S\right)\neq T$, go to step (2). Otherwise, $N_{G_{l}^{0}}\left(S\right)=T$. Find
  (A16) Δ=min(l(u)+l(v)≥w(u,v)|u∈S,v∈V−T)
  and replace existing labeling *l* with $l^{{}^{\prime }}$ by
  $$l^{{}^{\prime }}\left(u\right)=\left\{\begin{array}{cc} l(u)-\Delta , & u\in S\\ l(u)+\Delta , & u\in T\\ l(u), & \mathrm{otherwise}. \end{array}\right.$$

The process continues until an equal subgraph consisting a complete match is obtained.

### Appendix B.2. Complexity of the K-M Algorithm

Theoretically, the K-M algorithm is guaranteed to reach the global optimal solution [47]. It is the first proved polynomial algorithm and it was observed to be a strongly polynomial by Munkres in Reference [48]. Further, Edmonds and Karp in Reference [49] noticed that it can be modified to $O(n^{3})$ running time.

In our case in each of the steps (1) or (2) the algorithm adds one edge to the matching and this happens O(|V|). In addition, it needs O(|V|) to find the right vertex (if there is one) and improving the labelling takes O(|V|) time to find delta and to update the labelling accordingly. We might have to improve the labelling O(|V|) times until we matching occurs. This makes for a total of $O(|V^{2}|)$. In all, there are O(|V|) iterations each taking O(|V|) work, leading to a total running time of $O(|V^{3}|)$.
